# An allosteric interleukin-1 receptor modulator mitigates inflammation and photoreceptor toxicity in a model of retinal degeneration

**DOI:** 10.1186/s12974-020-02032-8

**Published:** 2020-11-27

**Authors:** Rabah Dabouz, Colin W. H. Cheng, Pénélope Abram, Samy Omri, Gael Cagnone, Khushnouma Virah Sawmy, Jean-Sébastien Joyal, Michel Desjarlais, David Olson, Alexander G. Weil, William Lubell, José Carlos Rivera, Sylvain Chemtob

**Affiliations:** 1 Department of Pharmacology and Therapeutics, McGill University, Montreal, QC Canada; 2 Departments of Pediatrics, Ophthalmology, and Pharmacology, Hôpital Maisonneuve-Rosemont Research Center, 5415 Boul L’Assomption, Montreal, QC H1T 2 M4 Canada; 3 Hôpital Sainte Justine Research Centre, Montreal, QC Canada; 4 Department of Obstetrics & Gynecology, University of Alberta, Edmonton, AB Canada; 5 Department of Neurosurgery, Hôpital Sainte Justine, Montreal, QC Canada; 6 Department of Chemistry, University of Montreal, Montreal, QC Canada

**Keywords:** Retinal degeneration, Photoreceptors, cell death, Inflammation, Inflammasome, Interleukin-1, Interleukin-1 receptor, Rytvela

## Abstract

**Background:**

Inflammation and particularly interleukin-1β (IL-1β), a pro-inflammatory cytokine highly secreted by activated immune cells during early AMD pathological events, contribute significantly to retinal neurodegeneration. Here, we identify specific cell types that generate IL-1β and harbor the IL-1 receptor (IL-1R) and pharmacologically validate IL-1β’s contribution to neuro-retinal degeneration using the IL-1R allosteric modulator composed of the amino acid sequence rytvela (as well as the orthosteric antagonist, Kineret) in a model of blue light–induced retinal degeneration.

**Methods:**

Mice were exposed to blue light for 6 h and sacrificed 3 days later. Mice were intraperitoneally injected with rytvela, Kineret, or vehicle twice daily for 3 days. The inflammatory markers F4/80, NLRP3, caspase-1, and IL-1β were assessed in the retinas. Single-cell RNA sequencing was used to determine the cell-specific expression patterns of retinal *Il1b* and *Il1r1.* Macrophage-induced photoreceptor death was assessed ex vivo using retinal explants co-cultured with LPS-activated bone marrow–derived macrophages. Photoreceptor cell death was evaluated by the TUNEL assay. Retinal function was assessed by flash electroretinography.

**Results:**

Blue light markedly increased the mononuclear phagocyte recruitment and levels of inflammatory markers associated with photoreceptor death. Co-localization of NLRP3, caspase-1, and IL-1β with F4/80^+^ mononuclear phagocytes was clearly detected in the subretinal space, suggesting that these inflammatory cells are the main source of IL-1β. Single-cell RNA sequencing confirmed the immune-specific expression of *Il1b* and notably perivascular macrophages in light-challenged mice, while *Il1r1* expression was found primarily in astrocytes, bipolar, and vascular cells. Retinal explants co-cultured with LPS/ATP-activated bone marrow–derived macrophages displayed a high number of TUNEL-positive photoreceptors, which was abrogated by rytvela treatment. IL-1R antagonism significantly mitigated the inflammatory response triggered in vivo by blue light exposure, and rytvela was superior to Kineret in preserving photoreceptor density and retinal function.

**Conclusion:**

These findings substantiate the importance of IL-1β in neuro-retinal degeneration and revealed specific sources of *Il1b* from perivascular MPs, with its receptor *Ilr1* being separately expressed on surrounding neuro-vascular and astroglial cells. They also validate the efficacy of rytvela-induced IL-1R modulation in suppressing detrimental inflammatory responses and preserving photoreceptor density and function in these conditions, reinforcing the rationale for clinical translation.

**Supplementary Information:**

The online version contains supplementary material available at 10.1186/s12974-020-02032-8.

## Introduction

Photoreceptor cell death is a hallmark of many retinal degenerative disorders including age-related macular degeneration (AMD) [1–4], the leading cause of vision loss in the aging populations of developed countries [5]. Two types of AMD are clinically recognized: wet AMD, distinguished by choroidal neovascularization, and dry AMD, characterized by the degeneration of the retinal pigmented epithelium (RPE) and photoreceptors. Wet AMD is generally treated with anti-vascular endothelial growth factor therapies [6], while no therapy is currently available for dry AMD.

Substantial evidence points to inflammation as a key player in photoreceptor cell loss in retinal degenerative disorders including dry AMD [4, 7–13]. Accumulation of mononuclear phagocytes (MPs), a family of cells which includes monocytes, macrophages, and microglia, has been associated with photoreceptor damage in retinal degeneration [12, 14–18]. Under normal physiological conditions, the subretinal space and photoreceptor layer are devoid of MPs. Conversely, in pathological conditions, MPs are activated, invade the outer and subretinal space, and secrete pro-inflammatory cytokines, including tumor necrosis factor-α, interleukin (IL)-6, and, importantly, IL-1β, which is considered to play a major role in retinal degeneration [11, 19].

High levels of IL-1β are detected in the eyes of AMD patients [20] and the same applies to models of macular degeneration wherein IL-1β is generated by microglia/macrophages [21–23]. The NOD-like receptor family pyrin domain-containing 3 (NLRP3) inflammasome is the major mediator of IL-1β production in the retina [24, 25] and is required to activate caspase-1 which catalyzes the processing of pro-IL-1β into active IL-1β [26, 27]. IL-1, in turn, primes the assembly of NLRP3 and further augments inflammatory cytokine production via positive feedback [28]. Previous studies have shown that some components of drusen and the C1q complement protein can activate the NLRP3 inflammasome in macrophages [25] and promote the secretion of IL-1β [29]; the same applies with lipofuscin when it accumulates with age [30]. Once activated, the IL-1β signal amplifies inflammation in rodent eyes by inducing expression of Chemokine (C-C motif) ligand 2 (*Ccl2*), Chemokine (C-X-C motif) ligand 1 (*Cxcl1*), and C-X-C motif chemokine ligand 10 (*Cxcl10*), which recruit leukocytes [21]. Concordantly, transcriptomic analysis of human retinas afflicted with AMD reveals upregulation of the same chemokines [31].

However, opposing observations on the role of IL-1β in retinal degeneration have been reported. For instance, pronounced sub-retinal inflammation and photoreceptor degeneration are observed in pigmented *Cx3cr1*-null mice subjected to light exposure; under these conditions, IL-1 receptor antagonist (IL-1Ra) is protective against photoreceptor cytotoxicity [32]. Similar conclusions have been drawn regarding the role of IL-1β in photo-oxidative damage to photoreceptors in rats [21]. Concordantly, IL-1β can induce apoptosis of retinal endothelium [33], neuronal precursor cells [34], and photoreceptors, but the precise mechanism of photoreceptor death in retinal degeneration remains unclear [22, 23, 32, 35]. On the other hand, LaVail [36] demonstrated protective effects of IL-1β on photoreceptors, while others failed to observe preservation of photoreceptors by IL-1Ra in light-exposed rodents [37]. Hence, collectively, the role of IL-1β in photoreceptor degeneration is unclear and suggests possible opposing effects of IL-1β based on concentrations which can depend on cell types and presumed locations relative to targeted photoreceptors, as well as by genetic determinants including those that apply to the diverse signaling of the IL-1 receptor (IL-1R) resulting in neurodegeneration. Correspondingly, the specific identity of cells producing IL-1β and harboring the IL-1 receptor has not been clearly defined.

IL-1β can bind to two types of receptors, IL-1 receptor I (IL-1RI) and IL-1 receptor II (IL-1RII). The binding of IL-1 to IL-1R1 induces a conformational change that allows the binding of the IL-1 receptor accessory protein (IL-1RacP) to IL-1R1, which then triggers the inflammatory cascade [38]. IL-1RII acts as a decoy receptor as it binds IL-1 but does not transduce IL-1 signaling [39]. There are a limited number of anti-inflammatory therapies that target IL-1 signaling; this includes anakinra (commercially known as Kineret), a recombinant form of IL-1Ra that binds competitively to IL-1R and non-selectively blocks downstream signaling [40]. We had previously developed a novel all-D peptide allosteric modulator with the amino acid sequence rytvela, which is an intramolecular peptide derived from the sequence of IL-1RacP [41]. Intramolecular peptides function on the basis of disrupting protein-protein interactions [42], such as the interaction between IL-1R and IL-1RacP in the case of rytvela (remote from the IL-1β binding site), and may confer benefits such as functional selectivity, which is associated with improved efficacy and reduced side effects [43].

In the present study, we pharmacologically validate the role of IL-1β in a light-induced model of subretinal inflammatory photoreceptor degeneration [12, 18]. We hereby clarify using single-cell RNA sequencing–specific sources of IL-1β from perivascular MPs, and IL-1RI expression primarily on astroglia and vascular cells. We also show that rytvela (as well as the molecularly and pharmacologically distinct Kineret) inhibits inflammation and preserves photoreceptor density and function to a greater extent than Kineret in this model.

## Materials and Methods

### Animals

All animal experiments were approved by the Maisonneuve Rosemont Hospital Animal Care Committee and were performed in accordance with the Association for Research in Vision and Ophthalmology Statement for the Use of Animals in Ophthalmic and Visual Research. Male CD-1 mice (Charles River Laboratories, Kingston, NY) of 12–16 weeks of age were used in this study. We favored using these outbred mice since inbred mice may not reflect genetic diversity as observed in the human population. In addition, genetic background does not appear to contribute significantly to the variation observed in measurements of phenotype [44]. Moreover, CD-1 mice are less susceptible to spontaneous lesions affecting the eye, which makes them a good model for pharmacological and long-term safety studies for ocular phenotypes [45]. CD-1 mice were housed with a 12-h light/dark cycle (100–200 lux) with water and normal diet food available ad libitum*.*

### IL-1 receptor antagonists

Kineret (Sobi, Biovitrum Stockholm, Sweden) was supplied as a 150 mg/mL solution and diluted to the required concentrations for all experiments with phosphate-buffered saline (PBS). Rytvela (Elim Biopharmaceuticals, Hayward, CA) was supplied as a lyophilized powder, dissolved in PBS, and diluted to the concentrations required for the experiments described below.

### Blue light exposure model

Mice were dark-adapted overnight, then pupils were dilated using topical atropine (Alcon) prior to light exposure. Mice were exposed to blue light from a light-emitting diode (wavelength 450 nm, Apluschoice) at a light intensity of 6,000 lux for 6 h and then returned to regular conditions under a standard 12-h light/dark cycle until sacrifice on day 3 post-illumination. Mice were intraperitoneally injected with IL-1 receptor antagonists Kineret (4 mg/kg/12 h) or rytvela (1.5 mg/kg/12 h), or with phosphate-buffered saline (PBS) twice a day starting on the day of illumination until day 3. These doses were determined from our previous study [33]. Subsequently, the mice were euthanized on day 3 by cervical dislocation under isoflurane anesthesia, and eyes collected.

### Retinal section preparation

Eyes were enucleated and fixed in 4% paraformaldehyde (PFA) for 1 h at room temperature and then rinsed twice with PBS. The cornea and lens were gently removed from the eye. Posterior eyecups were kept in 30% sucrose overnight and then frozen in optimal cutting temperature (OCT) medium. Sections of the entire retina along the optic nerve were cut into 10-μm sagittal sections. Retinal sections were treated at room temperature for 1 h with a blocking solution consisting of 10% fetal bovine serum, 0.1% Triton X-100, and 0.05% Tween-20 in PBS. Retinal sections were then incubated overnight with primary antibodies, 1:200 fluorescein-labeled peanut agglutinin (PNA) (FL-1071) or 1:100 *Griffonia simplicifolia* Lectin I (GSL I) isolectin B4, Fluorescein (FL-1201) at 4 °C. The antibodies used were 1:100 NLRP3 (Abcam, ab91413), 1:100 IL-1β (Abcam, ab9722), 1:100 caspase-1 (BioVision, 3019), glial fibrillary acidic protein (GFAP) (Dako, Z0334), and IL1R1 (Santa Cruz, sc-393998). The retinal sections were then washed thrice with PBS and incubated with a secondary antibody solution consisting of 1% bovine serum albumin (BSA), 0.1% Triton X-100, 0.05% Tween-20, 1:500 Alexa Fluor donkey 594 anti-rat (A21209, Invitrogen), and 1:500 Alexa Fluor 488 goat anti-rabbit (A11070, Invitrogen) for 2 h at room temperature. Retinal sections were washed thrice with PBS and then flat-mounted onto glass slides with coverslips and Fluoro-Gel mounting medium (Electron Microscopy Sciences, Hatfield, PA). Sections were imaged using a laser scanning confocal microscope (Olympus IX81 with Fluoview FV1000 Scanhead) with the Fluoview Software at 30X magnification. The integrated density of GFAP was determined using ImageJ (National Institutes of Health, Bethesda, MD, USA). The integrated density is the area above the threshold for the mean density minus the background.

### Measurement of photoreceptor layer thickness

Fourteen measurements per central retinal section (with optic nerve) were performed at defined distances from the optic nerve. Analysis of outer nuclear layer thickness was performed using ImageJ. The area under the curve was integrated using Prism version 7.0A (GraphPad software).

The length of the photoreceptor cone segments was measured using ImageJ, with 4 to 6 measurements made per central retinal section.

### Retinal flat mount preparation

Eyes were enucleated and fixed in 4% PFA for 1 h and then rinsed twice with PBS. The neuroretina was carefully separated from the RPE/choroid/sclera complex and processed for immunostaining. Retinas were treated at room temperature for 1 h with a blocking solution consisting of 1% BSA, 1% normal goat serum, 0.1% Triton X-100, and 0.05% Tween-20 in PBS. The retinas were then labeled overnight at 4 °C with gentle shaking using the following primary antibodies: 1:400 anti-F4/80 (ab6640, Abcam) and 1:500 anti-IL-1β (ab9722, Abcam). The retinas were then washed thrice with PBS and incubated with a secondary antibody solution consisting of 1% BSA, 0.1% Triton X-100, 0.05% Tween-20, 1:500 Alexa Fluor donkey 594 anti-rat (A21209, Invitrogen), and 1:500 Alexa Fluor 488 goat anti-rabbit (A11070, Invitrogen) for 2 h at room temperature. Retinas were washed thrice with PBS and then flat-mounted onto glass slides with coverslips and Fluoro-Gel mounting medium (Electron Microscopy Sciences, Hatfield, PA). For MP quantification, images were captured and stitched together using the MosaiX feature of Axiovision 4.

### Quantification of activated MPs in the subretinal space

F4/80-stained cells were counted on flat mounts with photoreceptor segments facing the objective. Cell numbers were expressed as the mean number of F4/80^+^ cells per mm^2^.

### Single-cell RNA sequencing analysis

The *Mus musculus* wild-type retina single-cell RNA sequencing datasets were obtained using the accession numbers GSM3854512, GSM3854514, GSM3854516, and GSM3854518 and analyzed using the Seurat R package [46]. Cells having a unique feature count between 100 and 4000, a total molecule number of less than 10000, and a mitochondrial RNA percentage of less than 25 were included. A global scaling normalization method, LogNormalize, was employed. This function normalizes the feature expression measurements for each cell by the total expression, multiplies this by a scale factor (10,000 by default), and log-transforms the result. After normalization, the scale expression (*z*-scores for each gene) was calculated for downstream dimensional reduction. After integration using the Seurat alignment procedures, the integrated matrix was then used for downstream analysis and visualization. Principal component analysis was run on the scaled integrated data and the results of dimensionality reduction were visualized with Uniform Manifold Approximation and Projection (UMAP). The clusters obtained were annotated using the markers provided by Heng et al. [47]. The expression of the genes *Il1r1* and *Il1b* were illustrated using dot plots.

The *Mus musculus* immune single-cell RNA sequencing datasets were obtained using the accession number GSE126783 and analyzed using the Seurat R package [46]. Light-damaged and control Cx3cr1^YFP+^ cells were filtered based on unique feature counts and mitochondrial counts. Cells having a unique feature count of less than 4000 and a mitochondrial RNA percentage of less than 10 were kept. The 2 Seurat Objects were then merged. An integration was performed on this object following the Integration and Label Transfer Vignette (SATIJA LAB). The integrated matrix was then used for downstream analysis and visualization. Principal component analysis was run on the scaled integrated data and the results were visualized with UMAP. The clusters obtained were annotated using the markers provided by O’Koren et al. [48]. All the microglia sub-clusters were regrouped together, and 3 different cell types were annotated: microglia, perivascular macrophages (pv MFs), and monocyte-derived macrophages (mo MFs). The expression of the genes *Il1r1* and *Il1b* were illustrated using dot plots.

### Terminal deoxynucleotidyl transferase dUTP nick end labeling assay

TUNEL staining was performed according to the manufacturer’s protocol (In Situ Cell Death Detection Kit; Roche Diagnostics). Briefly, retinal flat mount or retinal sections were fixed in 4% PFA for 30 min and washed in PBS. Flat mounts or sections were then incubated for 90 min at 37 °C with the reaction mixture and the reaction was stopped by washing with PBS. Nuclei were stained with 4′, 6-diamidino-2-phenylindole (DAPI, Sigma, St. Louis, MO, USA). Images were captured with a laser scanning confocal microscope (Olympus IX81 with Fluoview FV1000 Scanhead) with the Fluoview Software at 30X magnification.

### Isolation of bone marrow–derived monocytes

Bone marrow–derived monocytes (BMDMs) were harvested from 12- to 16-week-old CD-1 mice sacrificed by cervical dislocation. Total mononuclear cells were flushed from femurs and tibiae with PBS. to subsequently isolate the BMDM population. Briefly, the suspension was centrifuged at 1500 rpm for 10 min. The supernatant was discarded, and the pellet was resuspended in Dulbecco’s Modified Eagle Medium (DMEM) supplemented with 10% FBS (085-150, Wisent Bioproducts), 1% penicillin and streptomycin, and 0.125 μg/mL macrophage colony-stimulating factor (M-CSF) (576406; Biolegend). The suspension of BMDMs was filtered using sterile cell strainers (40 μm; 352340; Corning) and seeded in 24-well plates. The culture medium was renewed every 2 days for 1 week.

### BMDM and retinal explant incubation

BMDMs were treated or not with 50 ng/mL lipopolysaccharide (LPS) (*Escherichia coli* 10004557; Thermos Fisher) for 24 h and then 1 mM of adenosine triphosphate (ATP; R0441; Thermo Fisher Scientific) was added for 30 min. BMDMs were washed twice with DMEM supplemented with 0.2% BSA (800-095-EG; Wisent). BMDMs were cultured for 24 h and the supernatant served as conditioned media. Mouse retinas were prepared and placed on either 100,000 adherent BMDMs for 18 h at 37 °C in DMEM with 0.2% BSA or in BMDM-derived conditioned media in the absence or presence of rytvela (1 μM), Kineret (1.5 mg/mL), or an anti-IL-1β antibody (150 ng/mL, Abcam 9722). Doses of rytvela and Kineret were determined based on our previous study [49]. After 18 h, the explants were evaluated by TUNEL assay.

### Quantitative RT-PCR

Total RNA was extracted from mice retinas using the RNeasy mini kit (Qiagen) according to the manufacturer's protocol and was reverse transcribed using iScript™ Reverse Transcription Supermix (Bio-Rad) according to the manufacturer’s guidelines to generate cDNA. qPCR reactions were performed using 25 ng of sample cDNA, 2 μM of specific primers for the selected mRNAs (Table 1), and Universal SYBR Green Master Mix (BioRad). Relative expression (RQ = 2^−ΔΔCT^) was calculated using a detection system (ABI Prism 7500, Applied Biosystems, Foster City, CA, USA) and normalized to β-actin (*Actb*) and 18S.

**Table 1:** List of primers used for qPCR experiments

Gene	Forward primer	Reverse primer
*Il1b*	5′-CTGGTACATCAGCACCTCACA-3′	5′-GAGCTCCTTAACATGCCCTG-3′
*Il6*	5′-ACAGAAGGAGTGGCTAAGGA-3′	5′-AGGCATAACGCACTAGGTTT-3′
*Ccl2*	5′-CCACAACCACCTCAAGCACT-3′	5′-AGGCATCACAGTCCGAGTCA-3′
*Actb*	5′-GTGGGCCGCACAAGGCACCAA-3′	5′-CTCTTTGATGTCACGCACGA-3′
*18S*	5′-CCTGCGGCTTTAATTTGACTCA-3′	5′-AGCTATCAATCTGTCAATCCTGTC-3′

### Western blotting

Proteins were extracted from mice retinas by sonication in lysis buffer RIPA buffer (pH = 8) containing 50 mM Tris-HCl, 150 mM NaCl, 5 mM EDTA, 1% Triton 100×, 0.5% sodium deoxycholate, 0.1% SDS, and a cocktail of protease and phosphatase inhibitors (MiniComplete, PhosphoStop, and PMSF, Roche, Bâle, Switzerland). Protein concentrations were determined using the Bicinchoninic Acid Protein Assay Kit (Pierce, Rockford, IL, USA). Thirty micrograms of protein per sample were electrophoresed on 15% sodium dodecyl sulfate-polyacrylamide gels using an electrophoresis system (Mini-Protean Tetra System, Bio-Rad, Hercules, CA, USA) and then transferred onto polyvinylidene difluoride membranes (Millipore, Billerica, MA, USA). Membranes were blocked with 5% skim milk in Tris-buffered saline containing 0.1% Tween-20 for 1 h at room temperature, and incubated with the following primary antibodies: 1:800 IL-1β (Abcam, ab9722), 1:500 caspase-1 (BioVision, 3019), and 1:1000 β-actin (Santa Cruz, sc47778) then incubated with 1:6000 horseradish peroxidase-conjugated secondary antibodies (Millipore, AP307P and Millipore, AP308P). Densitometric analysis of western blotting bands was quantified using the ImageJ software and normalized to β-actin.

### Enzyme-linked immunosorbent assay

Conditioned media from BMDMs incubated with retinal explants were collected. The concentrations of IL-1β were measured using a commercial ELISA kit (R&D Systems, MLB00C) according to the manufacturer’s instructions. Calibration curves were prepared using purified standards for IL-1β which were provided as part of the ELISA kit.

### Electroretinogram

Electroretinograms (ERGs) were recorded using an Espion ERG Diagnosys apparatus equipped with a ColorDome Ganzfeld stimulator (Diagnosys LLC, Lowell, MA). Mice were initially dark-adapted overnight and anesthetized intraperitoneally with a mix of ketamine (100 mg/kg) and xylazine (20 mg/kg). Corneas were anesthetized with proxymetacaine hydrochloride (0.5% Alcaine; Alcon, Fort Worth, TX, USA) and pupils dilated with 0.5% atropine (Alcon, Fort Worth, TX, USA). Body temperature was maintained at 37.5 °C with a heating pad. ERGs were measured using corneal DTL Plus electrodes (Diagnosys LLC), a forehead reference electrode, and a ground electrode subcutaneously in the tail. To evaluate rod photoreceptor function (scotopic ERG), five-strobe flash stimuli were presented with flash intensities of 0.01 candela*second/meter^2^ (cds/m^2^), 0.1 cds/m^2^, 0.5 cds/m^2^,1.0 cds/m^2^, and 3.0 cds/m^2^. Six waveforms were averaged per light intensity. All procedures were performed in a dark room under dim red-light illumination. The amplitude of the a-wave was measured from baseline to the primary negative peak, and b-wave was measured from the trough of the a-wave to the maximum of the fourth positive peak.

### Statistical analysis

Values are presented as means ± standard error of the mean (SEM). Data were analyzed by independent t-tests or one-way analysis of variance (ANOVA) followed by post hoc Holm-Sidak tests for comparison of means. Statistical significance was set at *p* < 0.05. Statistical analysis was performed using Prism 7.0A (GraphPad Software).

## Results

### Blue light induces subretinal macrophage infiltration, which is suppressed by IL-1R antagonism

**Fig. 1:**
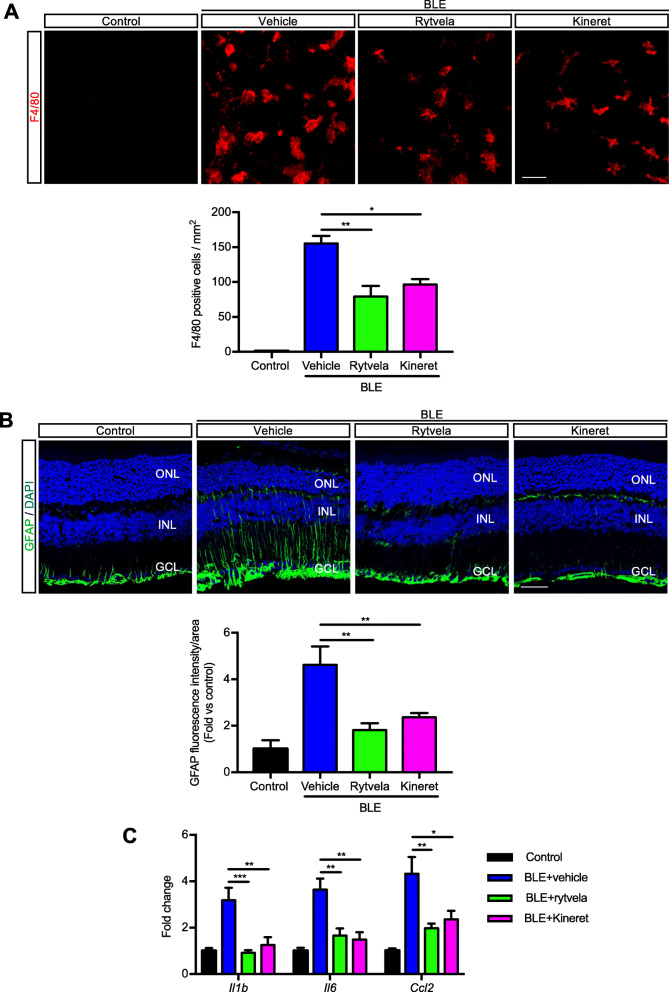
Subretinal macrophage infiltration and gliosis in blue light exposure to mice. **a** Representative images of retinal flat mounts showing infiltration of F4/80-labeled mononuclear phagocytes (red) of mice exposed or not to blue light exposure (BLE) and treated with vehicle, rytvela, or Kineret. Scale bar 50 μm. The graph represents compiled data on F4/80^+^ cell density in the subretina presented as a histogram. Data are expressed as mean ± SEM and analyzed by one-way ANOVA with Holm-Sidak correction for multiple comparisons; *n* = 4–8 per group. **p < 0.01 *p < 0.05. **b** Representative images of GFAP immunoreactivity (green) showing retinal gliosis in blue light-exposed animals treated with vehicle, and suppressed by rytvela and Kineret. Sections were co-stained with DAPI (blue) to show cell nuclei. Scale bar 50 μm. ONL: outer nuclear layer; INL: inner nuclear layer; GCL: retinal ganglion cell layer. The graph represents the quantitative analysis of GFAP immunofluorescence intensity compared with control light unexposed values set at mean of 1. Data are expressed as mean ± SEM and analyzed by one-way ANOVA with Holm-Sidak correction for multiple comparisons for *n* = 3–6 per group. **p < 0.01 (C) mRNA expression of *Il1b*, *Il6*, and *Ccl2*, standardized to control light unexposed values set at mean of 1. Data are expressed as mean ± SEM and analyzed by one-way ANOVA with Holm-Sidak correction for multiple comparisons for *n* = 3–6 per group. ***p < 0.001, **p < 0.01, *p < 0.05

**Fig. 2:**
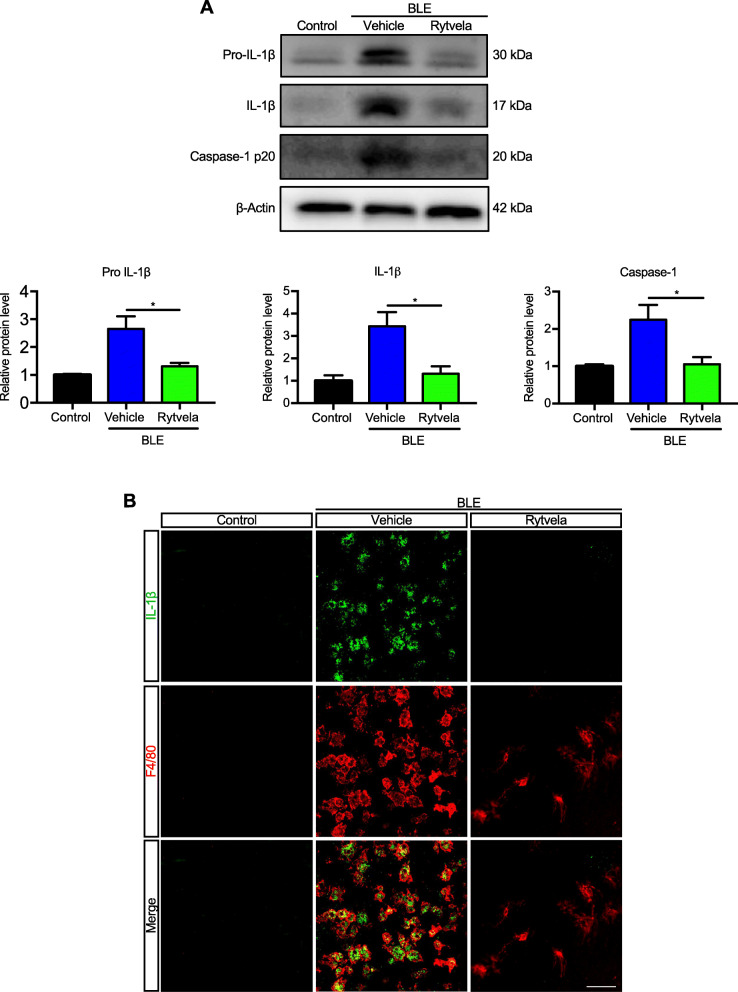
IL-1β production in blue-light-exposed mice. **a** Representative western blots (top panel) showing the expression of uncleaved pro-IL-1β, mature IL-1β, and cleaved caspase-1 p20 in retinal samples from blue light-exposed animals untreated (vehicle) or treated with rytvela and compared with non-illuminated animals (control). The bottom panel depicts compiled data in histogram format. Data are expressed as mean ± SEM and analyzed by independent *t* tests; *n* = 3–6 per group. **p < 0.01, *p < 0.05. **b** Representative images of retinal flat mounts showing co-localization of IL-1β (green) in mononuclear phagocytes F4/80^+^ cells (red) from blue light–exposed animals treated with vehicle or rytvela. Mice exposed to blue light and treated with rytvela displayed less IL-1β immunoreactivity; retinal samples from non-illuminated animals showed no positive reaction. *n* = 6 per group. Scale bar 50 μm

**Fig. 3:**
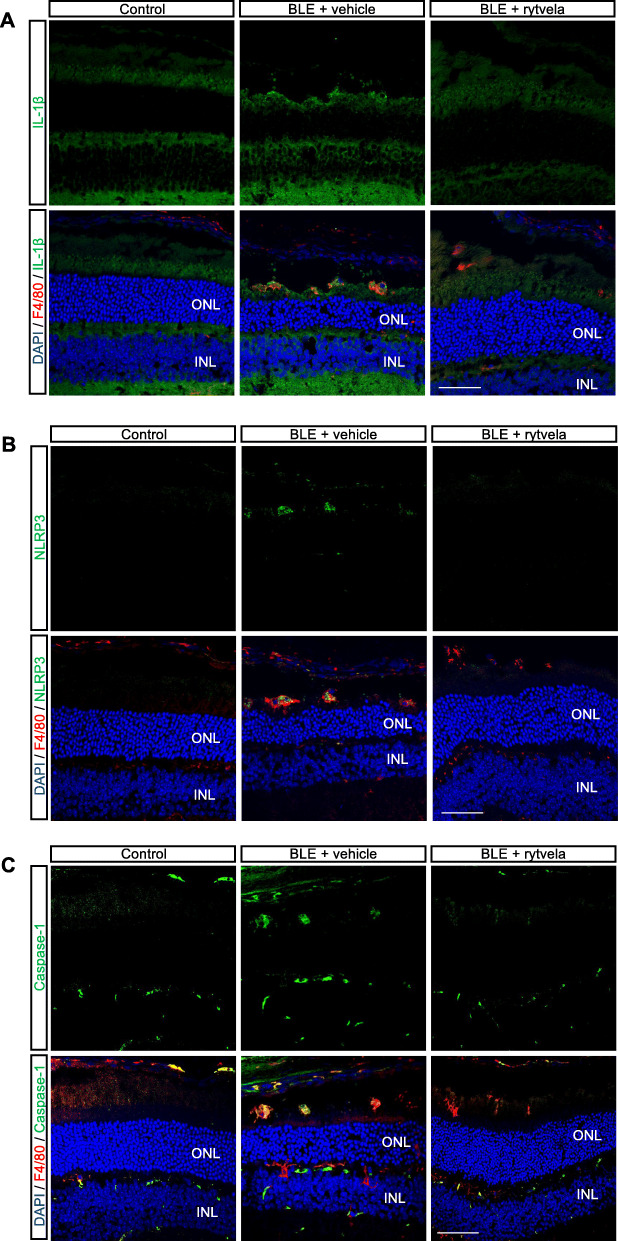
Subretinal distribution of IL-1β, inflammasome (NRLP3), and caspase-1 after blue light exposure (BLE). Representative confocal images showing co-immunoreactivity of **a** IL-1β (green), **b** NLRP3 (green), and **c** caspase-1 (green) with MPs F4/80^+^ cells (red) in the subretinal space of animals non-exposed (Control) and exposed to blue light (BLE) treated or not with rytvela. Cell nuclei were counterstained with DAPI (blue). Rytvela reduced immunoreactivity of IL-1β, NLRP3, and caspase-1. *n* = 4**–**5 per group. Scale bar 50 μm. ONL: outer nuclear layer; INL: inner nuclear layer

Blue light elicited substantial infiltration of MPs as evidenced by the presence of F4/80^+^ cells in the outer and subretina (Fig. 1a), consistent with reported observations [12, 18]. MPs are not seen in the subretinal space of unexposed (intact) retinas. Kineret, and to a slightly greater extent, rytvela mitigated this MP infiltration and maintained MP morphology in a ramified, quiescent state, rather than the activated ameboid state seen in vehicle-treated animals (Fig. 1a). Glial fibrillary acidic protein (GFAP), a marker of retinal gliosis [50, 51] which is upregulated upon MP activation [52] and used as an index of retinal degeneration, was dramatically increased throughout most of the entire retina (Fig. 1b); this effect was largely prevented by rytvela and Kineret. Concordantly, light-induced increases in pro-inflammatory *Il1b*, *Il6*, and *Ccl2* mRNA levels were also significantly reduced by the IL-1R inhibitors rytvela and Kineret (Fig. 1c). Similar observations were made for inflammasome components and products, whereby increased blue light–induced NLRP3, caspase-1, and IL-1β at different stages of maturation (Fig. 2a) co-localized in F4/80^+^ MPs (Figs. 2b and 3). Of relevance, retinal flat-mount staining revealed that IL-1β largely co-localized with MPs (Fig. 2b), suggesting that infiltrating MPs are the major source of this pro-inflammatory cytokine.

### Single-cell RNA-seq analysis of *Il1b* and *Il1r1* expression in the retina

**Fig. 4:**
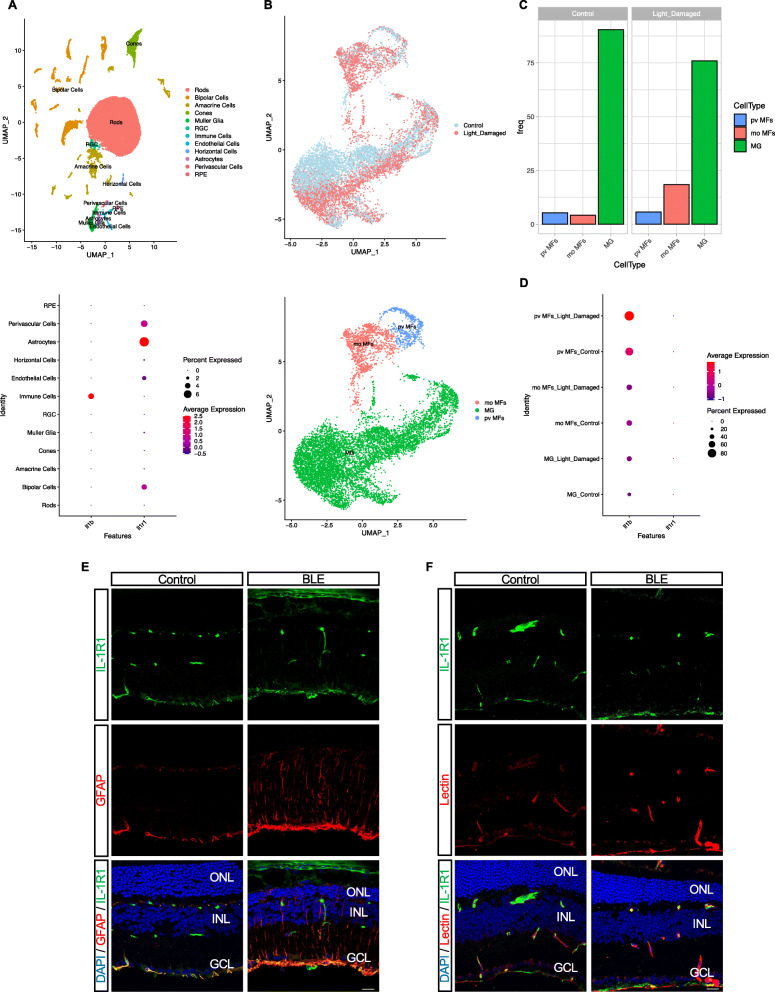
Expression of *Il1b* and *Il1r* genes across retinal cell clusters. **a** UMAP plot of droplet-based single-cell RNA sequencing (scRNA-seq) data obtained using 10× Genomics technology and representing retinal cell type from adult mouse retina (*n* = 4, 10–16 weeks). The plot shows a two-dimensional representation of global gene expression relationship among 33942 cells clustered into 12 retinal cell types (top panel). The expression levels of *Il1r1* and *Il1b* are represented as dot plots across all the 12 cell types; larger dots indicate broader expression within the cluster; deeper red denotes a higher expression level (bottom panel). **b** UMAP plot of droplet-based scRNA-seq data obtained using 10× Genomics technology showing different clusters (top panel). UMAP plot of droplet-based single-cell RNA sequencing (scRNA-seq) data obtained using 10× Genomics technology and representing retinal cell types from control and light-damaged (LD) adult mouse retina. scRNA-seq data were generated on fluorescence-assisted cell sorting (FACS)-sorted live Cx3cr1^YFP+^ cells from pooled neuroretinas of normal (*n* = 5) and LD (*n* = 8) mice. MG: microglia; pv MFs: perivascular macrophages; mo MFs: monocyte-derived macrophages. The plot shows a two-dimensional representation of global gene expression relationship among 10582 cells clustered into 3 retinal cell types (bottom panel). **c** Bar plot representation of Cx3cr1^YFP+^ cell proportions in different conditions. **d** The expression levels of *Il1r1* and *Il1b* are represented as dot plots across all the 3 immune cell types; larger dots indicate broader expression within the cluster; deeper red denotes a higher expression level. Representative confocal images showing co-immunoreactivity of IL-1R1 (green) with **e** GFAP^+^ cells (red) in the ganglion cell layer and **f** lectin (red). *n* = 4 per group. Scale bar 20 μm. ONL: outer nuclear layer; INL: inner nuclear layer; GCL: ganglion cell layer

We analyzed the transcriptomic profiles of *Il1b* and its receptor *Il1r1* in the mouse retina. Single-cell mRNA transcriptomic analysis using 10× Genomics revealed that immune cells are the main producers of *Il1b* in the retina and that *Il1r1* is mostly expressed in vascular cells (pericyte and endothelial cells) as well as astrocytes and bipolar cells (Fig. 4a). More importantly, *Ilr1* expression was not detected in photoreceptors and MPs (Fig. 4a). We next analyzed the proportions of different FACS-sorted live *Cx3cr1* MPs in control and light-damaged retinas. Among the 3 different cell types identified (Fig. 4b), light-damaged retinas showed a higher proportion of mo MF cells compared with the non-illuminated retinas (Fig. 4b, c), while microglia and pv MF distributions remained relatively unaffected. However, we found that *Il1b* was only upregulated in pv MFs but not in mo MFs or microglial cells in light-challenged mice compared with control (Fig. 4d). Expression of IL-1R1 was confirmed by immunofluorescence, showing that it colocalizes with GFAP and IB4 lectin in astrocytes and blood vessels, respectively (Fig. 4e, f), but not in MPs (Suppl Fig. 1).

### Suppression of subretinal inflammation preserves photoreceptor integrity

**Fig. 5:**
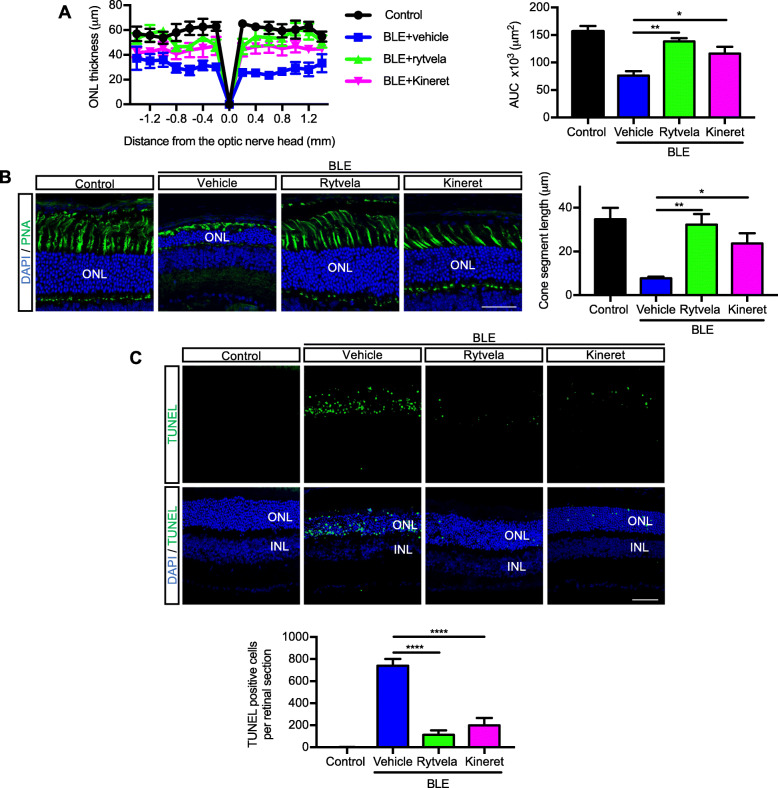
Prevention of photoreceptor cell death by the IL-1R modulator rytvela. **a** Spider-graph quantification of ONL thickness on DAPI-stained retinal sections from non-illuminated animals (control) and from blue light–exposed mice treated with vehicle, rytvela, or Kineret. Statistical analysis was performed using the area under the curve values (to assess photoreceptor density). Data are expressed as mean ± SEM and analyzed using one-way ANOVA with Holm-Sidak correction for multiple comparisons; *n* = 4–5. **p < 0.01, *p < 0.05. ONL: outer nuclear layer; AUC: area under the curve. **b** Representative images of photoreceptor cone outer and inner segments using fluorescein-PNA (green)-stained retinas. Scale bar 50 μm. The graph illustrates the quantitative analysis of cone segment length. **c** Representative images of TUNEL (green)-stained retinas in control and blue light-exposed mice administrated with vehicle or rytvela. The graph illustrates the quantitative analysis of TUNEL-positive cells in the ONL. Scale bar 50 μm. Data are expressed as mean ± SEM and analyzed using one-way ANOVA with Holm-Sidak correction for multiple comparisons; *n* = 6 per group. ****p < 0.0001

**Fig. 6:**
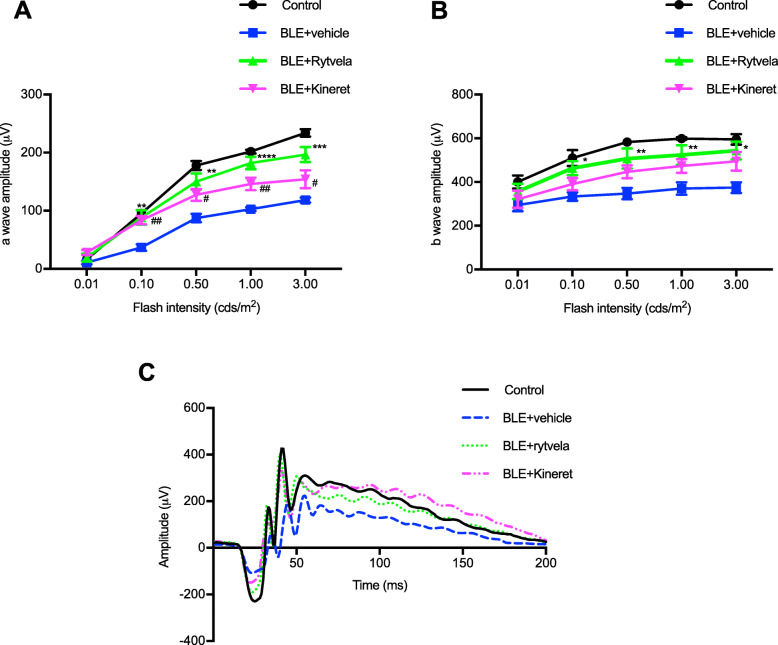
Preservation of photoreceptor function by the IL-1R modulator rytvela in light-exposed mice. Animals were exposed to blue light and treated as described in Fig 1. Flash intensities of 0.01, 0.1, 0.5, 1, and 3 cds/m^2^ were used in ERG analysis. Quantification of **a** a-wave amplitude and **b** b-wave amplitude of control, and blue light–exposed (BLE) mice treated or not with rytvela or Kineret. Data are expressed as mean ± SEM and analyzed using one-way ANOVA with Holm-Sidak corrections for multiple comparisons; *n* = 6–7 per group. ****p < 0.0001, ***p < 0.001, **p < 0.01 rytvela compared with vehicle. ##*p* < 0.01, #*p* < 0.05 Kineret compared with vehicle. **c** Representative waveforms of the electroretinogram recording at the flash intensity 3 cds/m^2^ from non-illuminated animals (control) and from blue light–exposed mice treated with vehicle, rytvela, or Kineret

Next, we evaluated whether the suppression of subretinal inflammation using rytvela or Kineret was associated with preservation of photoreceptor integrity. Mice exposed to blue light experienced significant photoreceptor degeneration evidenced by a thinner outer nuclear layer (ONL; Fig. 5a), a loss of cone inner and outer segments (evaluated by peanut agglutinin [PNA] staining; Fig. 5b), high apoptotic rate in the ONL corroborated by augmented TUNEL positivity (Fig. 5c), and associated loss of the a-wave ERG amplitude generated from the photoreceptors (Fig. 6a). At an optimal flash intensity of 3.00 cds/m^2^, the a-wave amplitude of light-exposed animals decreased by half compared with the control group; as expected, b-wave amplitude was also lower in vehicle-treated animals since it is triggered in bipolar and Müller cells by the a-wave signal (Fig. 6b). Rytvela and Kineret prevented apoptosis (Fig. 5c, d) and preserved photoreceptor layer thickness, including its outer segments (Fig. 5a, b), and photoreceptor function, with rytvela apparently being more effective than Kineret (Figs. 5a, b; and 6).

### Rytvela protects against macrophage-induced photoreceptor cell death in an ex vivo model

**Fig. 7:**
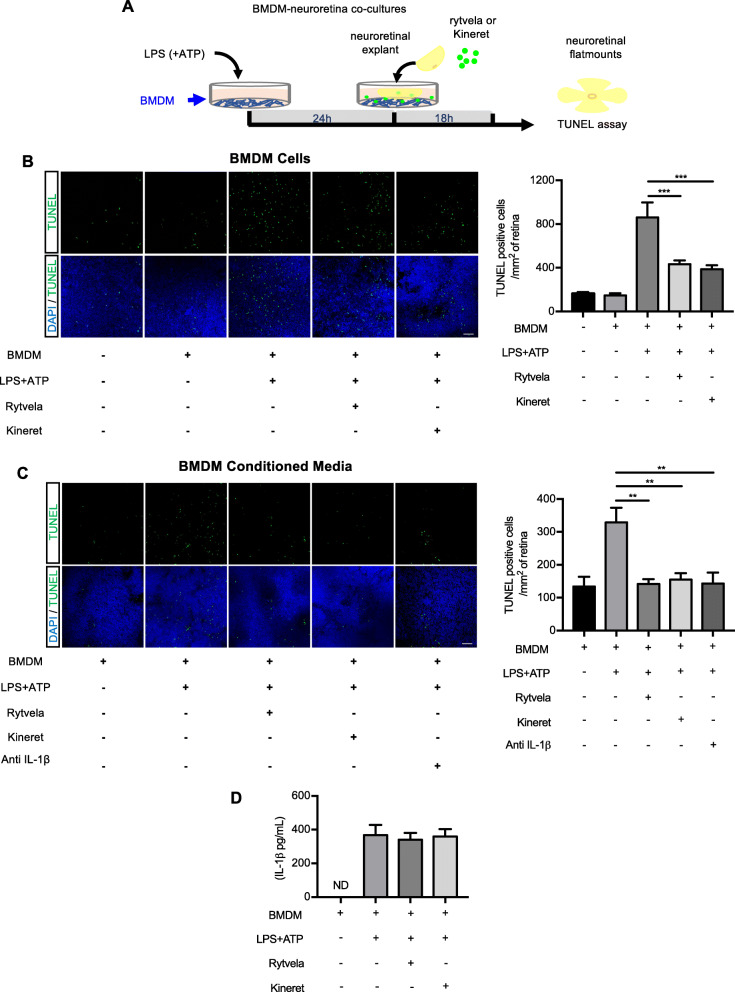
Effects of IL-1R inhibition (using rytvela and Kineret) on BMDM-induced photoreceptor toxicity. **a** An illustration of the experimental design used to evaluate the effects of isolated murine bone marrow-derived MPs (BMDMs) stimulated with LPS/ATP (stimulant of IL-1β secretion). **b** TUNEL-stained retinal flat mounts cultured in contact with BMDMs for 18 h in the presence or absence of rytvela or Kineret. Scale bar 50 μm. The graph represents the quantification of TUNEL-positive nuclei in the ONL of retinal flat mounts. Data are expressed as mean ± SEM and analyzed by one-way ANOVA with Holm-Sidak correction for multiple comparisons; *n* = 5–6 per group. ***p < 0.001. **c** TUNEL-stained retinal flat mounts cultured with the conditioned medium of LPS/ATP-activated or not BMDMs in the presence or absence of rytvela, Kineret, or an anti-IL-1β antibody. Scale bar 50 μm. The graph represents the quantification of TUNEL-positive nuclei in the ONL of retinal flat mounts. Data are expressed as mean ± SEM and analyzed by one-way ANOVA with Holm-Sidak correction for multiple comparisons; *n* = 3–5 per group. **p < 0.01. **d** ELISA measurement of IL-1β in the conditioned medium derived from BMDMs treated or not with LPS + ATP. The conditioned medium was incubated with retinal explants for 18 h in the presence of vehicle, rytvela, or Kineret. *n* = 4–5 per group. ND: not detected

We next proceeded to clarify if inflammation triggered by inflammasome activators, notably LPS, causes photoreceptor cell death, and if inhibition of IL-1β would prevent the latter. To evaluate this mechanism, we induced a pro-inflammatory phenotype in isolated murine BMDMs stimulated with LPS/ATP (which also stimulates formation of mature IL-1β [53]; Fig. 7a). LPS/ATP-stimulated BMDMs were incubated facing the photoreceptor layer of neuroretinal explants in the presence or absence of the IL-1R antagonists rytvela (1 μM) or Kineret (1.5 mg/mL) (Fig. 7a). Photoreceptor apoptosis was quantified by measuring TUNEL-positive cells in retinal explants. Exposure of retinal explants to LPS/ATP-stimulated BMDMs caused pronounced apoptosis of photoreceptors as witnessed by high numbers of TUNEL-positive cells (Fig. 7b); rytvela and Kineret prevented LPS/ATP-induced photoreceptor cell death. The number of TUNEL-positive cells did not differ between retinal explants cultured in the absence of BMDMs and the presence of non-activated BMDMs. In order to determine whether IL-1R inhibition in the neuroretina has a protective effect on photoreceptors, we treated neuroretinas with conditioned medium from BMDMs stimulated or not with LPS/ATP. Conditioned media from LPS/ATP-stimulated BMDMs caused an increase in the number of TUNEL-positive cells that was prevented by treating the neuroretina with rytvela or Kineret; these treatments did not affect the levels of IL-1β (Fig. 7c, d). To further clarify that IL-1β from activated BMDMs is involved, we used an IL-1β-neutralizing antibody which abrogated cell death (Fig. 7c). Taken together, these in vivo and ex vivo observations highlight the role of IL-1β and show that modulation of IL-1R signaling using rytvela (or IL-1R antagonist Kineret) interferes with detrimental subretinal inflammation in models of retinal phototoxicity and preserves photoreceptor density and function.

## Discussion

Neuroinflammation-induced photoreceptor cell death is a common feature of several ocular neurodegenerative disorders, including retinitis pigmentosa and AMD [54]. The recruitment and activation of MPs are key components in the progressive loss of photoreceptors in these types of ocular disorders [55, 56]. Several studies have suggested that pro-inflammatory mediators released by these infiltrating immune cells in the subretinal space are the main contributors to neuronal damage [57–59]. In this context, the major proinflammatory cytokine IL-1β has been suggested to promote acute neuronal loss in experimental studies and has been implicated in chronic neurodegenerative disorders [60–63], including those of the eye [11]. IL-1β levels are found to be increased in the vitreous humor of patients with AMD [20] and have been reported to contribute to photoreceptor degeneration in different animal models [21, 23, 32]. Yet, experimentally, a cytotoxic role for IL-1β has been questioned by others [36, 37], inferring possible distinct mechanisms including those based on IL-1β concentration and/or genetic determinants including those applied to the diversity in IL-1RI-coupled signaling processes. Moreover, the identity (and presumed location) of cells generating IL-1β and harboring IL-1RI remains to be clarified in order to better elucidate the role that IL-1β plays in retinal degeneration.

The present study used a blue light–triggered model of inflammation-induced photoreceptor toxicity, which is commonly utilized [12] to experimentally recapitulate the phenotypic changes observed in neuro-retinal degenerative disorders [64, 65]. We observed infiltration/activation of MPs associated with increased pro-inflammatory cytokines, including IL-1β and photoreceptor cell death. Essentially, pv MFs were the main source of elevated *Il1b* expression, whereas the receptor *Ilr1* was separately expressed on astroglia and vascular cells. The detrimental effects of light-induced inflammation were prevented by treatment with a negative modulator of IL-1R, to an extent superior to that observed with an orthosteric antagonist.

Under physiological conditions, the subretinal space hardly harbors any MPs due to immunosuppression mediated by inhibitory signals from neurons [66] and RPE cells [67]. However, MPs migrate to the subretinal space upon damage to the photoreceptors and/or RPE cells to scavenge cell debris in a process of efferocytosis which does not involve inflammatory responses [68]. With time upon exposure to light, exaggerated signals generated by trans-retinal Müller cells [55], RPE [69], and other MPs [56]. Activated MPs (revealed by their ameboid morphology, absence of cell processes, and inflammatory cytokine profile) can contribute to retinal gliosis wherein Müller cells further release factors that amplify the inflammatory response [70]. In this sense, we showed augmented GFAP immunoreactivity in Müller glia associated with subretinal MP infiltration after blue light exposure. Although one of the roles of Müller cells is to protect tissues from damage, their over-activation can contribute to neurodegeneration and curtail regenerative processes [71]; concordantly, GFAP gene knockout prevents gliosis and protects photoreceptors [72]. Relevantly, the IL-1R modulator rytvela suppressed MP activation and recruitment, minimized gliosis, and exerted neuroprotection.

MPs are reported to elicit their pathogenic effects at least in part through IL-1β [32]. We detected infiltrating MPs as the main source of IL-1β associated with photoreceptor phototoxicity. In support of the fact that the NLRP3 inflammasome is a key regulator of IL-1β maturation via the activation of caspase-1 [73], we observed the colocalization of the inflammasome components NLRP3, caspase-1, and IL-1β in infiltrating subretinal MPs (F4/80^+^). Our findings reveal that IL-1R antagonism suppresses IL-1β production as well as neuroretinal phototoxicity in cells that separately express IL-1β and IL-1RI. Rytvela prevented elevations in IL-1β levels after blue-light exposure mostly by reducing the accumulation of IL-1β-producing MPs into the subretinal space. It was also shown to reduce activation of glial cells (gliosis), which is reported to upregulate IL-1β production via ATP release and activate the inflammasome in a P2X7-dependent mechanism [74]; cytotoxicity is also known to release ATP (and high mobility group box 1 [HMGB1]) to stimulate IL-1β release by macrophages. Rytvela (and Kineret) in turn suppresses IL-1RI-dependent (photoreceptor) apoptotic processes involving mechanisms such as TRIF [75], IL-8 [76], glutamate transport [77], and reactive oxygen/nitrogen species [78]. Hence, one can envisage that light exposure triggers a macrophage chemotactic and activating signal (such as through release of DAMPs and chemokines) [79] which would generate IL-1β in MPs; IL-1β would be amplified by IL-1R signaling which can sustain activation of infiltrating MPs while also triggering cell death [32]. Antagonism of IL-1R (with rytvela or Kineret) would arrest this cycle and protect photoreceptors.

Since an important feature in this study applies to the fundamental role of infiltrated MPs that produce IL-1β, which contributes to photoreceptor degeneration, we specifically designed an experiment to show that activated BMDMs (known to generate high levels of IL-1β) or their conditioned media can trigger photoreceptor death in retinal explants, and is preventable by rytvela and Kineret, which act on IL-1RI-expressing neuroretina without reducing IL-1β concentrations in the BMDM conditioned media.

Using single-cell RNA-seq analysis, we validated that the long-lived pv MFs, which represent a small population of retinal MPs, were the main source of upregulated IL-1β. pv MFs can be defined by their location in contact or close association with the abluminal side of blood vessels [80]. Interestingly, these MPs can migrate from the perivascular space to the photoreceptor layer, participating in retinal degeneration [81].

Although subretinal MPs are implicated in photoreceptor toxicity via IL-1β secretion, one cannot rule out the involvement of other retinal cell types in modulating inflammation during retinal dystrophies. For instance, IL-1β secreted by infiltrating microglia/macrophages was shown to induce chemokine (Ccl2, Cxcl1, and Cxcl10) expression in Müller and RPE cells implicated in photo-oxidative retinal damage [21]. Conversely, IL-1β inhibition suppressed chemokine expression [21], consistent with our observations. In the mouse retina, we found that *Il1r1* expression was essentially in astrocytes, pericytes, endothelial cells, and bipolar cells. The failure to detect this receptor in MPs and photoreceptors suggests that IL-1β-induced neurotoxicity occurs through other cell type–specific IL-1R1 signaling pathways. One plausible mechanism of this effect might be in part due to the action of IL-1β on astrocytes to trigger glial activation/gliosis. We showed that IL-1R antagonism attenuates retinal gliosis, which is known to cause photoreceptor death. In addition, the action of IL-1β on astrocytes can result in the breakdown of blood-retinal barrier to increase vascular permeability which allows recruitment of MPs to the site of inflammation [82, 83]. Similarly, IL-1β can directly act on pericytes and endothelial cells to disrupt the integrity of blood-retinal barrier [84–87] which in turn, favors vascular permeability. Along these lines, the higher proportion of the short-lived mo MFs in the illuminated retinas suggests a possible disruption of the blood-retinal barrier to allow the infiltration of circulating monocytes [56]. Taken together, phototoxicity releases mediators that attract and stimulate macrophages at the site of injury, which amplify neuroretinal damage via activation of IL-1R. Additional studies are needed to further elucidate the distinct roles of IL-1R in different cell types.

IL-1β plays a complex role which also appears to be concentration-dependent, such that physiologic (low) levels of IL-1β confer protection against photoreceptor degeneration [36, 88]. Low levels of IL-1β enhance production of growth factors [89, 90] such as basic fibroblast growth factor (bFGF), which are relevant to vasculature and photoreceptor protection [21]. Conversely, pathologically elevated levels of IL-1β cause vascular decay [37, 91, 92]. Despite the evidence pointing toward an important function of IL-1 in photoreceptor death, a previous study has shown that *Ilr1* and *Casp1* deletion was not protective in a light-model of retinal degeneration [93]. However, these results with *Ilr1* knockout animals must be interpreted with caution since compensatory changes may have been adopted during development. In contrast, a more recent study showed that *Caspase-1/11* ablation has a protective role in retinal degeneration [94]. All in all, it is thus reasonable to propose that modulation of IL-1R would be a better alternative to (total) orthosteric antagonism of IL-1R, as the former would bias signaling and induce pharmacological selectivity, whereas the latter would inhibit all signals linked to IL-1R [95]. Along these lines, the allosteric modulator rytvela confers an advantage over orthosteric inhibitors of IL-1R by inhibiting the canonical JNK/p38/c-Jun/AP-1 pathway while preserving NF-κB, which is important for immune vigilance and photoreceptor survival [49, 96, 97]. Accordingly, rytvela protects the choroid and retina from inflammation without displaying adverse effects [33, 91, 98].

In summary, this study pharmacologically validates a major role for IL-1β in triggering neuro-retinal inflammatory responses and neural tissue damage in a model of light-exposed inflammation-triggered neurodegeneration. The development of pharmacologic allosteric IL-1R modulators represents a novel and promising therapeutic approach to tackle retinal degenerative diseases in which IL-1β is implicated. In this context, the advantages of the small peptide rytvela are its superior cytoprotective efficacy and small size compared with current anti-IL-1 therapeutics molecules conferring better distribution [49], ease of administration, low risk of immunogenicity [41], and maintenance of immune vigilance [97].

## Conclusion

Our study implicates an important role of IL-1β signaling in subretinal inflammation and photoreceptor death. We report astroglial and vascular expression of *Ilr1* and that *Il1b* is exclusively upregulated in pv MFs in response to light challenge. Additionally, the IL-1R modulator rytvela confers neuroprotection in photoreceptors following photooxidative stress to the retina. IL-1 receptor modulation is a promising therapeutic avenue to suppress the inflammatory response and preserve photoreceptor integrity in ocular degenerative diseases. It is hoped that our findings will pave the way for future investigations and culminate in clinical trials involving ophthalmic patient populations.

## Supplementary Information

**Additional file 1:** **Supplementary Figure 1.** The absence of colocalization of IL-1R1 with F4/80 in the neuroretina. Representative confocal images showing non-colocalization of IL-1R1 (green) with F4/80+ cells (red). *n* = 4 per group. Scale bar 20 μm. ONL: outer nuclear layer, INL: inner nuclear layer, GCL: ganglion cell layer.

## Data Availability

All datasets and analyses used in the current study are available from the corresponding author on reasonable request.
