# Supplementary figures and images for: An allosteric interleukin-1 receptor modulator mitigates inflammation and photoreceptor toxicity in a model of retinal degeneration
Source: J Neuroinflammation. 2020 Nov 27;17:359. doi: 10.1186/s12974-020-02032-8 (PMC7694438; doi:10.1186/s12974-020-02032-8)

Supplementary Figure 1

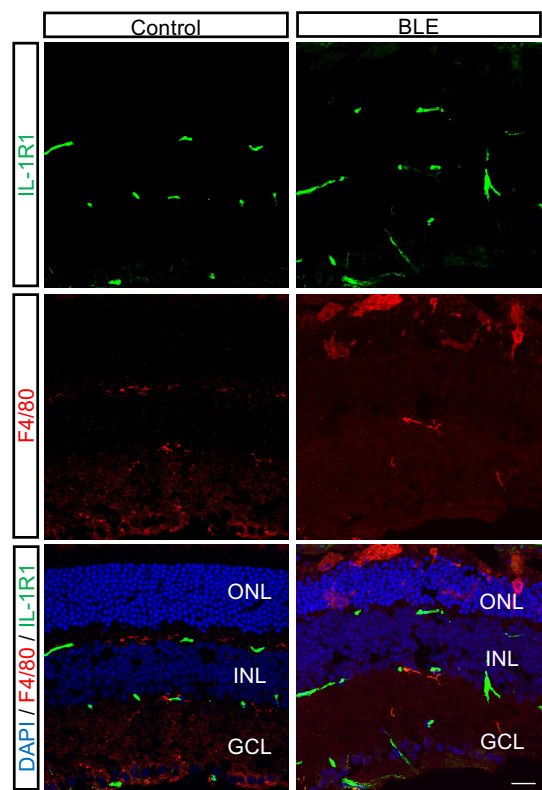

Supplement: Supplementary file 1 — Additional file 1: Supplementary Figure 1. The absence of colocalization of IL-1R1 with F4/80 in the neuroretina. Representative confocal images showing non-colocalization of IL-1R1 (green) with F4/80+ cells (red). n = 4 per group. Scale bar 20 μm. ONL: outer nuclear layer, INL: inner nuclear layer, GCL: ganglion cell layer. [file 12974_2020_2032_MOESM1_ESM.pdf]
